# Workspace Disorder Does Not Influence Creativity and Executive Functions

**DOI:** 10.3389/fpsyg.2018.02662

**Published:** 2019-01-15

**Authors:** Alberto Manzi, Yana Durmysheva, Shannon K. Pinegar, Andrew Rogers, Justine Ramos

**Affiliations:** ^1^School of Social and Behavioral Sciences, Mercy College, Dobbs Ferry, NY, United States; ^2^Borough of Manhattan Community College, City University of New York, New York, NY, United States; ^3^Baruch College, City University of New York, New York, NY, United States

**Keywords:** clutter, disorder, order, messy desk, creativity, divergent thinking, executive function

## Abstract

Recent research by Vohs et al. (2013) garnered media attention after reporting that disordered environments increase creativity. The present research was designed to conceptually replicate and extend this finding by exploring the effect of workspace disorder on creativity. Participants were randomly assigned to work at a neatly organized (Order condition) or a messy desk (Disorder condition), where they completed several paper-and-pencil and computerized tasks, including two validated creativity measures (Abbreviated Torrance Test for Adults; ATTA; Goff and Torrance, 2002; Alternative Uses Task; adapted from Guilford, 1967). We also included several executive control measures from the NIH EXAMINER (Kramer, 2011), to explore the role of reduced top-down control in explaining a possible creativity-disorder connection. Independent-samples *t*-tests failed to replicate any significant difference in creativity between the Order and Disorder conditions. Furthermore, the conditions did not differentially affect executive control. Despite implementing an experimental setup similar to the one in Vohs et al. (2013), including a larger sample size, and adopting multiple measures of the constructs of interest, we did not find any effect of workspace clutter on cognitive performance. At this stage, the relationship between disorder and cognition seems elusive and does not warrant the claims it generated in the popular press.

## Introduction

Creativity involves thinking in novel, original and useful ways, and can manifest itself in many different contexts and activities (Runco, 2004). Although traditionally creativity has been viewed as a product of internal characteristics of the creator, in recent years researchers dedicated efforts to studying environmental influences on creative processes (Amabile and Pillemer, 2012). Among them are Vohs et al. (2013), who explored the role of environmental disorder on creativity. In their study, college students were administered an adaptation of the Alternative Uses Task (AUT; Guilford, 1967), and asked to generate as many alternative/unusual uses as possible for a ping-pong ball while sitting at a desk scattered with papers and books (Disorder condition) or neatly organized (Order condition). Indeed, those in the Disorder condition generated ideas rated as 0.41 points more creative on a 3-point scale than those in the Order condition. They also generated more highly creative ideas (scored 3 on a 3-point scale), though the total number of ideas was equivalent in the two groups. The authors speculated that a cluttered environment could implicitly prime an attitude of “breaking with tradition” (p. 1860), whereas a neat environment would prime the concept of “playing it safe” (p. 1866).

Despite media coverage going as far as advocating for messy desks at work (Vohs, 2013; Hill, 2018), these findings have yet to be replicated and need scientific attention. Therefore, we set out to explore why and how physical disorder would improve creative thinking. We hypothesized that a possible explanatory key might reside in the relationship between creativity and executive functioning, and how the latter may be influenced by cluttered environments. Evidence suggests that reduced top-down control may facilitate divergent thinking by enabling access to a broader range of nodes in the semantic network and allowing bottom-up processing, which is more exploratory and less guided by cognitive schemas (Vartanian et al., 2007; Beaty et al., 2016). Research showed increased creativity following experimental manipulations aimed at reducing cognitive control via neurostimulation of the frontal cortex (Chrysikou et al., 2013), after exposure to an experimental ego-depletion paradigm using a taxing executive control task (Radel et al., 2015), after bodily activities that exhaust control resources (Zhou et al., 2017) or in contexts that facilitate mind wandering and “open focus” (Baird et al., 2012; Colzato et al., 2017). Furthermore, in correlational studies, White and Shah (2006, 2011) reported that individuals with ADHD struggled to inhibit irrelevant information yet outperformed non-ADHD individuals in divergent thinking tasks. Also, those who generated looser semantic associations to word pairs (possibly due to weaker top-down control) produced more inventive creations (White and Shah, 2016; see also, Zabelina et al., 2016, on the relationship between divergent thinking performance and “flexible” attention).

Are executive functions drained by environmental disorder? And could this explain the disorder-creativity connection? Psychophysical research indicates that cluttered visual environments increase reaction times and decrease executive control regardless of whether this is assessed concurrently or after exposure (McMains and Kastner, 2011; McCarley et al., 2012). Berman et al. (2008) found that a stroll through a busy city street lowered executive control and working memory compared to a quiet park, with similar results induced by looking at pictures of nature compared to urban environments (therefore in contexts that did not require engagement or interaction with the environment, but simply exposure). Furthermore, in designs similar to the study by Vohs et al. (2013), Chae and Zhu (2014) assigned participants to orderly or disorderly workstations, although with clutter displayed vertically, behind and above the desk. The Disorder condition performed worse at the Stroop task and gave up sooner on an unsolvable puzzle task. It can be speculated that disorderly environments offer distractions, which compete for attentional resources. Inhibiting these distractions would increase the demand on the executive control system; therefore, resulting in reduced cognitive control compared to tidy environments.

The present study is aimed at further investigating the effect of cluttered work environments on creativity and executive control. The goal was to conceptually replicate the clutter effect on creativity (Vohs et al., 2013) using another measure of creativity (the Abbreviated Torrance Test for Adults; ATTA; Goff and Torrance, 2002) in addition to the AUT. Concurrently, we tested the effect of clutter on different measures of executive control to explore the role of executive functioning in the disorder-creativity connection. We also included individual differences measures of personality and sensitivity to perceptual context (field dependence-independence), as these might help understand the differences in susceptibility to the effect of environmental disorder and serve to assure group equivalence.

## Methods

### Participants

One-hundred and fourteen volunteers participated in the study. After exclusions due to data corruption or non-compliance, a final sample of 100 was retained (Age: *M* = 22.89, *SD* = 5.19; 69% female; 81% right-handed; see demographics in Supplementary Material). Participants were compensated with a US$20 gift card or extra credit. The study was approved by Mercy College Institutional Review Board, and informed consent was obtained prior to entry into this study.

### Measures

The protocol included paper-and-pencil and computerized tests assessing personality, cognitive style, creative divergent thinking, and executive functions (for additional details, see Supplementary Material).

The NEO-Five Factor Inventory 3 (NEO-FFI-3; Mccrae and Costa, 2004) was used to assess the Big Five personality domains: Neuroticism, Extraversion, Openness, Agreeableness, and Conscientiousness. The Group Embedded Figures Test (GEFT; Witkin et al., 1971) measured field independence, the cognitive style related to visually perceiving an element as separate from its context.

The Alternative Uses Task (AUT; adapted from Guilford, 1967) was administered to measure divergent thinking and creativity, by asking participants to write up to 10 alternative/unusual uses for an automobile tire in 5 min. Inappropriate responses were rejected and identical/overlapping entries were coded as repetitions. Two raters blind to experimental condition assessed creativity holistically on a 5-point scale by considering commonality, remoteness and cleverness [see scoring directions in Silvia et al. (2008)]. To avoid that very similar responses were not assigned different creativity scores, the final pool of pertinent responses (referred to as “fluency” in the results’ tables) was sorted in homogenous categories (e.g., gardening, furniture, etc.) and then judged for creativity. Inter-rater reliability on creativity scores was high as scores assigned by the two raters were very highly correlated [Pearson’s *r* = 0.92; *p* < 0.001].

The Abbreviated Torrance Tests for Adults (ATTA; Goff and Torrance, 2002) was also administered to obtain additional measures of verbal and figural creativity. The first task (ATTA 1) asked participants to list possible problems associated with being able to fly or walk on air. Scoring followed the procedure outlined for the AUT and resulted in very high inter-rater correlation [*r* = 0.98; *p* < 0.001]. In the second task (ATTA 2) participants used two incomplete abstract figures to create two drawings and give them a title. Similarly, in the third task (ATTA 3) participants used 9 triangles arranged in a 3 × 3 matrix to draw pictures and title them. Creativity was scored as described above and reliability was good [*r* > 0.90; *p* < 0.001; *r* = 0.74, *p* < 0.001, respectively]. Overall, for all creativity tasks, the high inter-rater correlations indicated that scorers adopted similar criteria to assess creativity. To honor the slight differences in creativity ratings produced by the two raters, scores were averaged before data analysis.

To assess different aspects of executive functioning, the following NIH EXAMINER subtests (Kramer, 2011^1^) were used: *Letter Fluency* (L, F) and *Category Fluency* (animals, vegetables) in 1-min blocks; *Set Shifting*, requiring responding to the shape or the color of bivalent stimuli in homogenous or intermixed blocks of trials to compute “mixing costs” (RT difference between mixed-task blocks and single-task blocks) and “switching costs” (RT slowing when switching tasks in a mixed block; Monsell, 2003); *Flanker Task*, *Digit Span Task (verbal working memory)*, and *N-back Task (visuo-spatial working memory)* (see tasks descriptions and scoring criteria in Supplementary Material).

### Procedure

**FIGURE 1:**
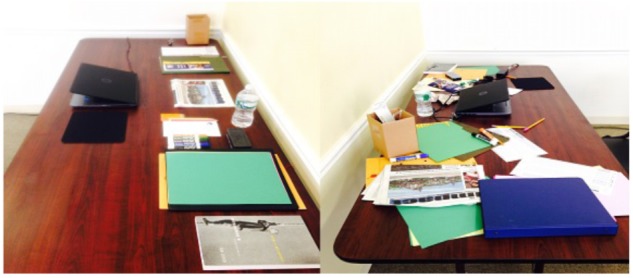
Workspace setup for the Order (left) and the Disorder (right) conditions.

Participants were tested individually in two adjacent identical classrooms at Mercy College in New York City. They were first placed in a neutral environment, identical for both conditions, to complete informed consent, personality and cognitive style measures. Then, they were directed to the workspace, where desk materials and chairs were arranged in an orderly or disorderly fashion (see Figure 1). Here, after a 3-min delay, the remaining tasks were administered. Task order was counterbalanced between participants with a 5-min break scheduled midpoint in the session. Upon completion, participants were debriefed and compensated.

## Results

Conditions were equivalent for demographic and individual differences of the participants (see Supplementary Material). Independent-samples *t*-tests were conducted to compare the creativity and executive function measures between the Order and Disorder conditions (see Table 1). A Bonferroni-corrected alpha-level was set at 0.0125, given the four creativity and four executive measures being compared, respectively. The number of titles produced at the ATTA 2, and the number of figures and titles for ATTA 3 returned the smallest *p*-values and largest effects (*p* = 0.09, *p* = 0.05, and *p* = 0.09, respectively; see Table 1). However, these trends should be interpreted with caution because they were isolated and greater than the adopted significance cut-off. No condition differences were found for the average creativity ratings for all creativity tasks, even when these values were collapsed in an overall creativity measure obtained by averaging the standardized scores for all four variables.

**Table 1:** Creativity and executive function measures, separately for the Order and Disorder conditions.

Variables	Order		Disorder		Statistics^1^	Cohen’s *d* [CI]^2^
				
	M (SD)	[CI]	M (SD)	[CI]		
**Alternative Uses Task (AUT)**						
Fluency	5.52 (2.78)	[4.72, 6.31]	6.32 (2.53)	[5.60, 7.04]	*t*(98) *=* -1.504, *p =* 0.14	0.30 [-0.09, 0.69]
Rejected responses	1.02 (2.00)	[0.45, 1.59]	0.90 (1.74)	[0.41, 1.39]	*t*(98) *=* 0.320, *p = 0*.75	-0.06 [-0.46, 0.33]
Repetitions	0.26 (0.63)	[0.08, 0.44]	0.30 (0.58)	[0.13, 0.46]	*t*(98) *=* -0.329, *p = 0*.74	0.06 [-0.33, 0.46]
Creativity average	2.80 (0.35)	[2.7, 2.90]	2.68 (0.47)	[2.55, 2.81]	*t*(92) *=* 1.365, *p = 0*.18	-0.29 [-0.68, 0.11]
Highly creative ideas	1.34 (1.29)	[0.97, 1.71]	1.10 (1.07)	[0.79, 1.41]	*t*(98) *=* 1.012, *p = 0*.31	-0.20 [-0.60, 0.19]
**ATTA – subtest 1**						
Fluency	4.84 (1.82)	[4.32, 5.36]	4.92 (2.06)	[4.33, 5.51]	*t*(98) *=* -0.206, *p = 0*.84	0.04 [-0.35, 0.43]
Rejected responses	0.90 (1.42)	[0.50, 1.30]	0.58 (1.20)	[0.24, 0.92]	*t*(98) *=* 1.220, *p = 0*.23	-0.24 [0.64, 0.15]
Repetitions	0.32 (0.54)	[0.17, 0.47]	0.30 (0.71)	[0.10, 0.50]	*t*(98) *=* 0.158, *p = 0*.86	-0.03 [-0.42, 0.36]
Creativity average	2.45 (0.84)	[2.21, 2.69]	2.55 (0.82)	[2.32, 2.78]	*t*(98) *=* -0.581, *p = 0*.56	0.12, [0.27, 0.51]
Highly creative ideas	1.64 (1.53)	[1.20, 2.08]	1.64 (1.340)	[1.26, 2.02]	*t*(98) *=* 0.000, *p =* 1.00	0.00 [-0.39, 0.39]
**ATTA – subtest 2**						
Fluency (figures)	1.74 (0.53)	[1.59, 1.89]	1.96 (0.81)	[1.72, 2.19]	*t*(98) *=* -1.614, *p = 0*.11	0.32 [-0.07, 0.71]
Fluency (titles)	1.50 (0.71)	[1.30, 1.70]	1.78 (0.91)	[1.52, 2.04]	*t*(98) *=* -1.718, *p = 0*.09	0.34 [-0.05, 0.74]
Creativity average	2.15 (0.13)	[2.11, 2.18]	2.33 (0.90)	[2.07, 2.58]	*t*(95) *=* -0.979, *p = 0*.33	0.28 [-0.12, 0.67]
Highly creative ideas	0.46 (1.03)	[0.17, 0.75]	0.66 (1.27)	[0.30, 1.02]	*t*(98) *=* -0.863, *p = 0*.39	0.17 [-0.22, 0.57]
**ATTA – subtest 3**						
Fluency (figures)	2.68 (2.20)	[2.05, 3.31]	3.56 (2.13)	[2.95, 4.17]	*t*(98) *=* -2.033, *p =* 0.05	0.41 [0.01, 0.80]
Fluency (titles)	2.48 (2.23)	[1.85, 3.12]	3.22 (2.06)	[2.64, 3.81]	*t*(98) *=* -1.721, *p = 0*.09	0.65 [-0.05, 0.74]
Creativity average	2.18 (0.89)	[1.92, 2.43]	2.26 (0.83)	[2.02, 2.50]	*t*(93) *=* -0.466, *p = 0*.64	0.09 [-0.30, 0.49]
Highly creative ideas	0.28 (0.70)	[0.08, 0.48]	0.26 (0.53)	[0.11, 0.41]	*t*(98) *=* 0.161, *p = 0*.87	-0.03 [-0.42, 0.36]
**Overall creativity average (z)**	**-0.03 (0.56)**	**[-0.19, 0.13]**	**0.00 (0.62)**	**[-0.17, 0.18]**	***t*(98) *=* -0.314, *p =* 0.75**	**0.06 [-0.33, 0.46]**
**Flanker task**						
Error difference	0.78 (2.10)	[0.18, 1.38]	0.78 (2.52)	[0.06, 1.50]	*t*(98) *=* 0.000, *p =* 1.00	0.00 [-0.39, 0.39]
Median RT Difference	0.16 (0.13)	[0.12, 0.20]	0.15 (0.11)	[0.12, 0.18]	*t*(98) *=* 0.423, *p =* 0.67	0.08 [-0.48, 0.31]
**Set-shifting task**						
Mixing cost median RT	0.28 (0.26)	[0.21, 0.35]	0.20 (0.23)	[0.13, 0.27]	*t*(97) *=* 1.629, *p =* 0.11	-0.33 [-0.72, 0.07]
Switching cost median RT	0.11 (0.12)	[0.08, 0.14]	0.10 (0.12)	[0.07, 0.13]	*t*(97) *=* 0.305, *p =* 0.76	-0.08 [-0.48, 0.31]
**Working memory**						
1-Back errors	2.78 (1.84)	[2.26, 3.30]	3.36 (2.22)	[2.73, 3.99]	*t*(98) *=* -1.421, *p =* 0.16	0.28 [-0.11, 0.68]
2-Back errors	23.58 (9.3)	[20.94, 26.22]	25.36 (10.5)	[22.3, 28.3]	*t*(91) *=* -0.863, *p =* 0.39	0.18 [-0.21, 0.57]
Dot-counting span	4.44 (1.30)	[4.07, 4.81]	4.02 (1.46)	[3.61, 4.43]	*t*(98) *=* 1.519, *p =* 0.13	-0.30 [-0.70, 0.09]
**Fluency**						
Letter – correct	21.96 (5.3)	[20.45, 23.47]	22.98 (7.2)	[20.9, 25.0]	*t*(90.1)^3^ *=* -0.806, *p =* 0.42	0.16 [-0.23, 0.55]
Letter – repetitions	0.14 (0.41)	[0.02, 0.27]	0.22 (0.58)	[0.06, 0.38]	*t*(98) *=* -0.798, *p =* 0.43	0.16 [-0.23, 0.55]
Letter – violations	0.36 (0.85)	[0.12, 0.60]	0.60 (1.03)	[0.31, 0.89]	*t*(98) *=* -1.270, *p =* 0.21	0.25 [-0.14, 0.65]
Category – correct	28.12 (6.7)	[26.22, 30.02]	29.18 (8.5)	[26.76, 31.60]	*t*(98) *=* -0.693, *p =* 0.49	0.14 [-0.25, 0.53]
Category – repetitions	0.54 (0.97)	[0.26, 0.81]	0.34 (0.96)	[0.07, 0.61]	*t*(98) *=* 1.034, *p =* 0.30	-0.21 [-0.60, 0.19]
Category – violations	0.62 (1.19)	[0.28, 0.95]	1.68 (3.46)	[0.70, 2.66]	*t*(60.5)^3^ *=* -2.05, *p =* 0.05	0.41 [0.01, 0.81]


*See Methods and Supplementary Material for details on the different performance indices; ^1^Different degrees of freedom are due to missing data for participants who did not complete a task. All ox*p*-values are reported for illustrative purposes; smallest oxp-values are underlined and discussed in the Results section; Bonferroni-corrected alpha was set at 0.0125; ^2^CI = 95% confidence interval computed using the ESCI software (https://thenewstatistics.com; Cumming, 2014); ^3^Welch’s correction applied due to equality of variances not assumed (significant Levene’s Test); ATTA, Abbreviated Torrance Test for Adults; RT, reaction times in milliseconds; Fluency – number of pertinent responses, figures, and/or titles produced in accordance to task instructions; Rejected responses – number of inappropriate responses; Repetitions – identical or overlapping responses produced by the same participant (and not included in the Fluency score); Highly Creative Ideas – number of ideas with creativity rating of 4 or 5 on a 5-point scale.*

All Order-Disorder comparisons were not significant also in the case of the executive functioning measures (see Table 1).

Results remained unchanged after removing outliers scores 3 SD away from the mean (*n* = 5).

### Ancillary Analysis

A series of additional analyses were conducted to test differences between task rotations. We also compared the effect of experimental conditions only considering the rotations that most closely resembled the task order in Vohs et al. (2013) and Chae and Zhu (2014). Finally, we explored possible interaction effects between condition and executive control. These results are discussed in the Supplementary Material available online.

## Discussion

Our design employed a multi-measure approach to conceptually replicate and extend previous findings showing increased creativity in messy workspaces (Vohs et al., 2013). We included verbal and figural creativity tasks, as well as various executive functioning tasks to investigate the possible role of reduced top-down control in explaining a possible creativity-disorder connection. We hypothesized that a cluttered work environment would interfere with executive functioning, reducing top-down control and increasing access to a broader semantic network; thus, allowing participants to generate more creative ideas. Moreover, in keeping with recent statistical guidelines for sound replication studies (Simonsohn, 2015), we employed a sample twice as large as the one in the study by Vohs et al. (2013). Results in our study indicated that working at a messy desk did not result in significantly and reliably different creativity or executive functioning performance. Despite being confident that our design was not underpowered, we can identify some limitations that might explain the lack of significant differences between the Order and the Disorder conditions. For example, Vohs et al. (2013) asked participants to develop alternative uses for a ping-pong ball, whereas we used an automobile tire; they used a 3-point creativity rating scale whereas we used a 5-point scale. Nevertheless, the literature abounds of multiple variations on objects used in the alternative uses tasks [e.g., brick, shoe, newspaper, paper clip; see also the original task instructions by Guilford (1967)] and the scoring protocol we followed has been widely investigated and does not warrant radically different results. Most importantly, we employed multiple creativity measures, covering the spectrum of verbal and figural creative abilities; therefore, it is compelling that none of the measures showed the expected effect. Similarly, we noted certain differences between our study and the one by Chae and Zhu (2014), who reported an effect of disorder on executive functioning (overall RT at the Stroop task) and perseverance (time spent working on an unsolvable puzzle). Indeed, the authors used a more salient environmental manipulation, with clutter being displayed on the wall above and behind the participant’s desk [compared to objects spread across the table in our case and in the study by Vohs et al. (2013)], which might have resulted in a more powerful disruption of top-down control. Additionally, in two of the studies by Chae and Zhu (2014), participants remained in the manipulated environment when completing surveys (therefore, for longer than the 3-min delay in our study) before measuring executive functioning/perseverance. Furthermore, they explicitly asked participants about their perception of the room organization, increasing participant attention to the environment and possibly making the manipulation more effective.

Sample size remains one compelling difference between our study and the studies cited above, as both studies only included 20 to 25 participant per group. Outliers have a greater influence on groups with a smaller sample, a problem that we tried to minimize by doubling our sample sizes per group (*n* = 50). Since the authors do not report outlier analyses or detailed descriptive statistics, we speculate whether their results would persist with a broader sample.

In addition to the differences in experimental design discussed above, our participants may also perform differently because they score differently on individual difference measures. Indeed, exploratory *post hoc* analysis (see Supplementary Material) found an interaction between condition and executive function, pointing to a small condition effect (better creativity in the disorder condition) for those with better executive control. We caution against over reliance on this finding, as only one of the several executive function measures reflected this effect. Yet, the trend supports Vohs et al. (2013), and we can concede that overall differences in executive functioning between the two samples could potentially explain the different results. Unfortunately, the original study by Vohs et al. (2013) did not include descriptive statistics on individual differences, therefore our alternative explanation remains speculative.

Finally, we failed to ask our participants about their perceived sensitivity to disorder or preference toward workspace tidiness. Participants’ aversive reaction to cluttered environment might cause disengagement and lead participants to implement less top-down control, which could provide an alternative mechanism to understand the effect of clutter on cognitive performance. Unfortunately, we lack data on how participants’ reaction to the disorder manipulation (or lack thereof) might have contributed to our results.

Overall, given the limitations reviewed above, we believe that future research on the creativity-disorder connection should use manipulations leading to different levels of executive control draining or interference, include groups with high/low executive functioning, as well as implement more salient, persistent, and interactive exposure to the manipulated environment. It would also be informative to measure the preference and tolerance for clutter and disorder of each participant.

Failed replications pose a great challenge to science, as they might lead to dichotomous interpretations, that either the original study was wrong, or that the replication was inaccurate. We prefer to avoid extreme verdicts. Yet, we are confident in concluding that, given the variety of measures used and the sample size of our study, the effect of working at a disorderly desk for a limited span of time – if true – is small and unreliable. At the current stage of the scientific inquiry on this topic, such elusive phenomenon does not warrant the sensationalistic claims it generated in the popular press.

## Author Contributions

AM and YD contributed to design, implementation, data collection, data analysis, and manuscript preparation. SP contributed to data analysis and manuscript preparation. AR and JR were responsible for data collection, data scoring, and assistance with manuscript preparation.

## Conflict of Interest Statement

The authors declare that the research was conducted in the absence of any commercial or financial relationships that could be construed as a potential conflict of interest.

## Footnotes

1 https://memory.ucsf.edu/examiner-assessments
