# Supplementary material for: Workspace Disorder Does Not Influence Creativity and Executive Functions
Source: Front Psychol. 2019 Jan 15;9:2662. doi: 10.3389/fpsyg.2018.02662 (PMC6340966; doi:10.3389/fpsyg.2018.02662)

Workspace disorder does not influence creativity and executive functions.

Alberto Manzi, Yana Durmysheva, Shannon K. Pinegar, Andrew Rogers, Justine Ramos

SUPPLEMENTARY MATERIAL

Methods

2.2 Details on Executive Functions Tasks (EXAMINER)

The NIH EXAMINER battery (Kramer, 2011) includes subtests that assess different facets of executive functioning (fluency, task-switching, updating/working memory). The overall administration time of the subtests we included is approximately 30 minutes.

In the *Letter and Category Fluency Tasks*, participants were asked to come up with as many words as possible beginning with the letter L, the letter F, belonging to the category "animal", and belonging to the category "vegetable" (1 minute for each subtask). Performance was assessed as number of correct responses (reflecting retrieval speed), as well as errors in the form of repetitions (of the same answer more than once) and violations of the task rules (for example, producing the name of a city instead of common names for letter fluency; or a food that is not a vegetable for category fluency).

In the *Set Shifting Task*, participants had to respond to the shape or the color of bivalent stimuli in homogeneous blocks of "pure" trials (that is, blocks in which the same task is repeated) and intermixed blocks, in which naming the shape or the color randomly alternate. In the latter case of intermixed tasks, on a given trial participants might either perform the same task as on the previous trial ("stay" trials) or switch to the other task ("switch" trials). Following the task-switching literature (Monsell, 2003), we used mixing costs and switching costs as performance indices. Specifically, mixing costs were computed as the difference between RTs on stay trials in mixed blocks and pure trials in single-task blocks. Mixing costs have been associated with different processes summarized under the term task-set updating, and reflecting the active maintenance of multiple task-sets in working memory. Switching costs were measured as the difference in performance between switch and stay trials in mixed blocks, and are believed to capture task-set reconfiguration.

In the *Flanker Task*, participants responded to the orientation of a central arrow that could appear as flanked by arrows pointing in the same direction of the target or in the opposite one. Performance was assessed by subtracting reaction times (RTs) in the congruent conditions from RTs in the incongruent condition (reflecting processes involved in solving the interference due to the presence of conflicting information).

In the *Digit Span Task (verbal working memory)*, participants counted strings of dots out loud, keeping in mind the total number of dots over consecutive trials and producing it at the end of the sequence; the longest sequence correctly held and retrieved on at least two trials was used as a performance indicator.

In the *N-back Task (visuo-spatial working memory),* participants had to keep in mind the location of squares appearing on a screen one at a time, and comparing the location of each square to 1 or 2 squares presented before it (1-back and 2-back tasks, respectively); performance was assessed counting the number of errors committed for the each version of the n-back task.

Results

3.1 Ancillary analysis

Reliability Analysis on Individual Differences Measures. The FFI and GEFT inventories resulted highly reliable. Each FFI scale included 12 items and the Cronbach's alpha values for each scale were: .81 (Neuroticism); .75 (Extraversion); .70 (Openness); .71 (Agreeableness); .84 (Conscientiousness).

The GEFT included 18 items and its alpha was .92.

Task Order. The individual differences measures were all administered at the beginning of the session and in the same order (FFI-GEFT). Instead, the experimental tasks were administered in four possible rotations to counterbalance the effect of task order. The four orders were as follows: Rotation 1: AUT-ATTA-Break-EXAMINER; Rotation 2: ATTA-AUT-Break-EXAMINER; Rotation 3: EXAMINER-Break-AUT-ATTA; Rotation 4: EXAMINER-Break-ATTA-AUT. To test the differences between rotations, a series of 2 (Condition: Order/Disorder) X 4 (Rotation: 1, 2, 3 & 4) ANOVAs were conducted on all the creativity and executive functioning measures. The only two rotation effects with lowest p-values are reported below. There was a significant difference in the Category Fluency Repetitions, F (3, 96) = 2.73, p = 0.05, η^2_p= 0.08, where Rotation 2 (M = 0.79, SE = 0.19) was significantly higher than Rotation 1 (M = 0.23, SE = 0.19) and Rotation 4 (M = 0.13, SE = 0.19). Rotation 3 (M = 0.62, SE = 0.19) was marginally higher than Rotation 4 (M = 0.13, SE = 0.19). There were also significant differences in Category Fluency Violations, F (3, 96) = 2.77, p = 0.05, η^2_p= 0.08, where Rotation 4 (M = 2.42, SE = 0.52) was significantly higher than Rotation 1 (M = 0.69, SE = 0.19) and Rotation 2 (M = 0.5, SE = 0.52) and marginally higher than Rotation 3 (M = 1.04, SE = 0.50). When the ANOVAs were rerun with outliers excluded, the only significant main effect that remained between the rotations were the Category Fluency Repetitions, F (3, 91) = 2.79, p = 0.05, η^2_p= 0.08, where Rotation 2 (M = 0.83, SE = 0.20) was significantly higher than the Rotation 1 (M = 0.25, SE = 0.19) and Rotation 4 (M = 0.09, SE = 0.20). Rotation 3(M = 0.56, SE = 0.19) was marginally higher than Rotation 4(M = 0.09, SE = 0.20).
Overall, given the isolated rotation effects on two minor performance aspects, and given that the rotations were collapsed, we believe that rotation differences did not bias our results.

Rotation 1. We examined differences between Order and Disorder in the task rotation that most closely matched the task order in the paper by Vohs et al. (2013) (n = 25), in which the Alternative Uses Task was administered right after participants were moved to the orderly or disorderly workspace. When comparing all the creativity measures between the conditions, no significant differences were found. The smallest p-value (p = .079) was returned for the number of rejected responses at the ATTA 1 (M = 1.00, SD = 1.35; M = .23, SD = .60, respectively), however this finding was isolated and greater than the Bonferroni-corrected significance cut-off (.0125).

Rotation 3 and 4. Similarly, we compared performance on all the executive function measures between conditions using only Rotation 3 and 4 (n = 50), in which EF tasks were administered right after participants were moved to the orderly or disorderly workspace. Indeed, in these two rotations, the executive function measures were collected before the creativity ones, possibly reducing the risk of task interaction, fatigue, etc. The lowest p-value was obtained for Category Fluency violations (Order: M = .64, SD = 1.11; Disorder: M = 2.76, SD = 4.59; t (26.8) = -2.12; p = .03). Another low p-value was found for Category Fluency repetitions (Order: M = .56, SD = .92; Disorder: M = .20 , SD = .41; t (33.2) = .36; p = .08). It is worth noting that both p-values where far from the Bonferroni-corrected cut-off and the effects where in opposite directions, with more errors in the Disorder conditions in the first variable and less in the second. Overall, we feel confident to conclude that in our design the lack of condition effects on executive control was not attributable to task order.

Power analysis and sample size. We ran a power analysis using G-Power for an independent samples t-test with an effect size of 0.81 (which was the highest reported effect size by Vohs et al., 2013), using expected power of 0.80, and an alpha of 0.05. Given these constraints, the estimated total sample size per group is 50. When estimating the sample size needed for a multiple regression for a large effect size and power of 0.80 using 12 predictors, the total sample size is 61 people. Moreover, we followed recent guidelines on sample size in the context of replication studies (Simonsohn, 2015), and we increased the sample size to at least

twice the size of the original study by Vohs et al. (2013), resulting in a final retained sample of 100 participants.

Regression Analyses. Finally, to assess whether executive functioning interacted with Order-Disorder to influence creativity, we regressed average creativity on the executive function and experimental condition variables. Furthermore, we added interactions between the condition and all executive function variables. The model did not significantly predict creativity [$R^2 = 0.14$, F (13, 80) < 1]. The only effect that remained significant when accounting for all other variables was the interaction between condition and switching cost (RT slowing when switching tasks in a mixed block) [$B = -.16$, $p = .038$, $B_{zero} = -.17$], where lower switching cost (better executive control) led to higher creativity scores in the Disorder condition (gradient of simple slope = -0.18, $p = 0.085$) compared to the Order condition (gradient of simple slope = 0.14, $p = 0.24$) [see Table B and Figure A, below]. All other variables and interactions were not significant [ts < 1.4, $ps > .16$].

SUPPLEMENTARY TABLES & CHARTS

Supplementary Table A. Demographic information and individual differences measures (collected before exposure to experimental manipulation), separately for the Order and Disorder conditions.

	**Order**	**Disorder**	**Statistics**[1]
	**Count**	**Count**	
Sex	32 f, 17 m	37 f, 13 m	χ^2 (1, N = 99) = .885, p = .35
Handedness	9 l, 38 r	5 l, 43 r	χ^2 (1, N = 96) = 1.44, p = .23
Race/Ethnicity	18(W), 6(B), 7(A), 11(H), 8(O)	19(W), 15(B), 4(A), 7(H), 5(O)	χ^2 (4, N = 100) = 6.28, p = .17
	**M (SD)**	**M (SD)**	
Age	22.52 (5.04)	23.24 (5.36)	t (96) = -.683, p = .50
Years of College Education	2.34 (1.51)	2.59 (1.82)	t (94) = -.735, p = .45
GPA	3.29 (.46)	3.37 (.43)	t (76) = -.770, p = .44
FFI - Neuroticism	24.42 (8.16)	24.04 (8.11)	t (98) = .234, p = .82
FFI - Extraversion	28.30 (6.51)	29.50 (6.75)	t (98) = -.905, p = .37
FFI - Openness	32.30 (7.59)	33.72 (6.20)	t (98) = -1.02, p = .31
FFI - Agreeableness	31.42 (5.87)	33.18 (6.58)	t (98) = -1.41, p = .16
FFI - Conscientiousness	34.10 (6.45)	33.90 (7.85)	t (98) = .139, p = .89
GEFT	9.14 (5.49)	8.32 (5.53)	t (98) = .744, p = .46

Notes: [1] Different degrees of freedom are due occasional missing data from demographic forms; FFI = Five Factors Inventory; GEFT = Group Embedded Figures Test; Abbreviations for Race/Ethnicity are: W = White, B = Black/African-American, A = Asian, H = Hispanic, O = Other.

Supplementary Table B. Regression analyses.

Predictors	ΔR^2	B	SE B	β	P	Partial*	Partial┼
Step 1 (N = 94)	0.08						
Constant		-0.05	0.06	0	0.36	0	0
Condition		-0.01	0.06	-0.02	0.87	-0.02	-0.02
Mixing Cost		0.01	0.07	0.03	0.83	0.02	0.02
Switching Cost		-0.05	0.07	-0.07	0.53	-0.07	-0.07
Letter Fluency Correct		0.11	0.06	0.21	0.07	0.19	0.19
Category Fluency Correct		0.03	0.06	0.05	0.69	0.04	0.04
N Back One		0.08	0.06	0.14	0.2	0.14	0.13
Flanker Task Mean		-0.06	0.07	-0.1	0.36	-0.1	-0.1
Step 2 (N = 94)	0.06						
Constant		-0.06	0.06	0	0.33	0	0
Condition		0.18	0.26	0.33	0.49	0.08	0.07
Mixing Cost		0.04	0.07	0.06	0.61	0.06	0.05
Switching Cost		-0.02	0.08	-0.03	0.82	-0.03	-0.02
Letter Fluency Correct		0.09	0.07	0.16	0.2	0.14	0.14
Category Fluency Correct		0.02	0.07	0.03	0.8	0.03	0.03
N Back One		0.04	0.07	0.08	0.54	0.07	0.06
Flanker Task Mean		-0.06	0.08	-0.1	0.4	-0.09	-0.09
Condition X Mixing Cost		-0.11	0.07	-0.19	0.12	-0.17	-0.16
Condition X Switching Cost		-0.16	0.08	-0.25	0.04	-0.23	-0.22
Condition X Letter		0.04	0.07	0.07	0.6	0.06	0.06
Condition X Category		-0.01	0.01	-0.35	0.46	-0.08	-0.08
Condition X N Back		0.03	0.07	0.05	0.71	0.04	0.04
Condition X Flanker		0	0.08	0	0.97	0	0

Notes: All variables are grand mean centered; β = standardized regression coefficients, B = unstandardized regression coefficients; * Shared contributions of the predictors; ┼ Unique contributions of the predictors.

Supplementary Figure A. Adjusted interaction for Switching Cost Reaction Times (RT) and Order-Disorder Condition for Overall Creativity z-score

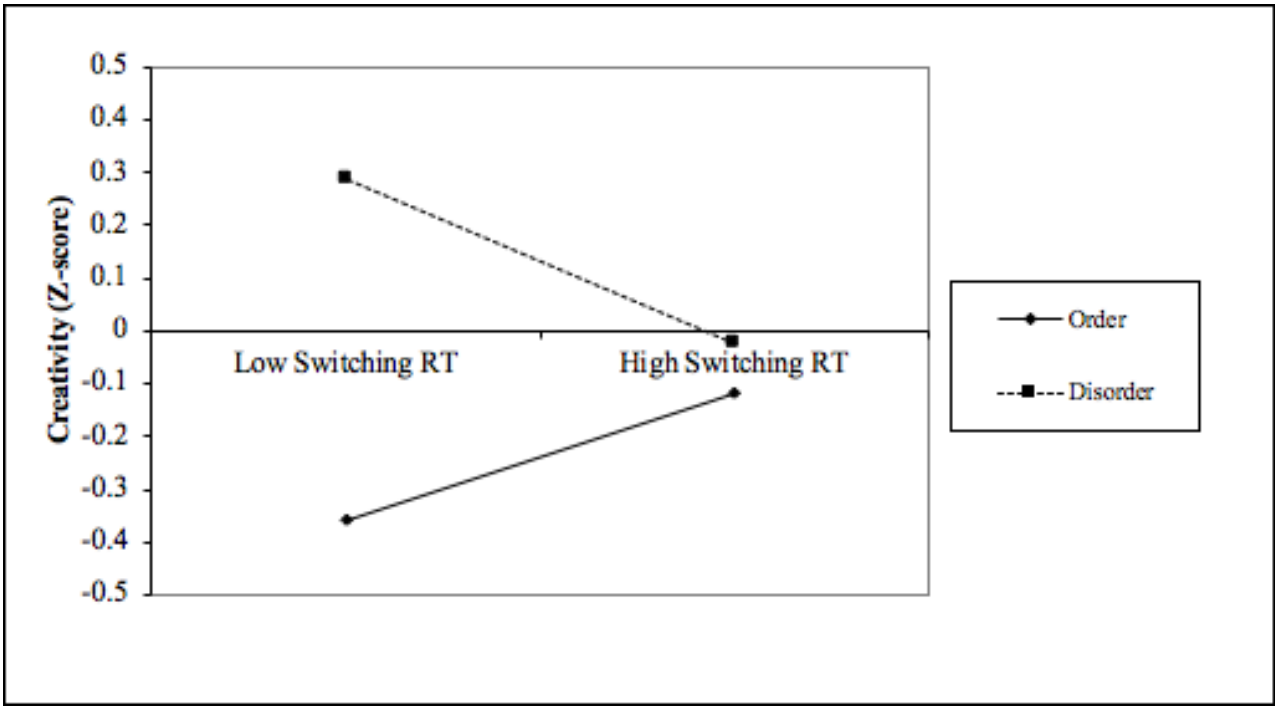

Supplement: Supplementary file 1 [file Presentation_1.pdf]
